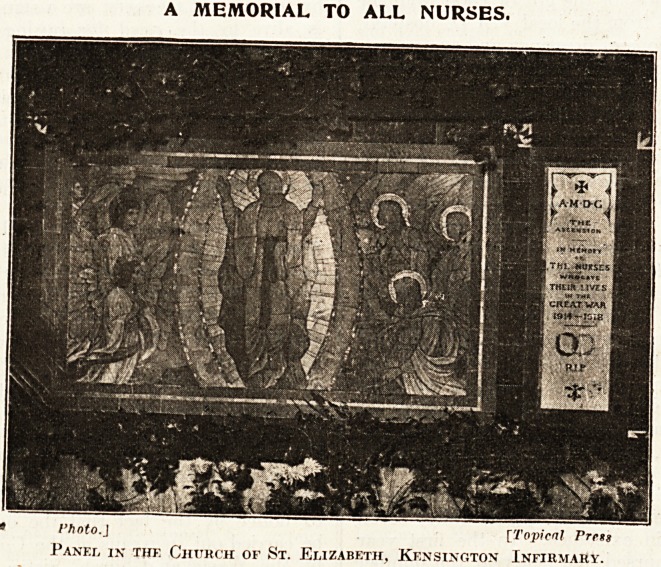# Round the Hospitals

**Published:** 1921-01-15

**Authors:** 


### ROUND THE HOSPITALS.

What is claimed to be the only general memorial to nurses who lost their lives in the course of duty during the war was unveiled by Mr. Thomas Stuttard on Thursday, January 6, the Feast of Epiphany, in the Church of St. Elizabeth, the beautiful little chapel in the grounds of the Kensington Infirmary. The memorial, which is reproduced on this page, is in the form of a panel, and is uniform with others which have been erected in this church from time to time. It is from the design of Mr. Wm. Glasby, and has been executed in mosaic by Simpson's. The cost, £100, has been met by the nurses of Kensington Infirmary and their friends in memory of all nurses who laid down their lives, and it is mainly to the Rev. A. Lombardini, Chaplain to the Infirmary, that the erection of the memorial is due. The Bishop of Croydon, in his address at the service on Thursday, dwelt on the sacrifice which had been gladly and freely rendered by nurses and warriors in the Great War, and, indeed, by all classes, a sacrifice which was made notable by the devotion which accompanied it and by the absolute confidence that right would prevail. After such a sacrifice there could be no return to the old conditions, and there was no room for pessimism regarding the future. The church was filled, and the music was beautifully rendered by the organist, Mr. W. H. Hickox, and a string orchestra under the direction of Miss Vickers, while the Kensington Infirmary nurses gave the carol "Christians, carol sweetly." A beautiful chaplet of arum lilies and palm leaves from the nursing staff of the infirmary was placed next to the Nurses' Panel.

### A MEMORIAL TO ALL NURSES.

*Photo.*] [*Topical Press*

Panel in the Church of St. Elizabeth, Kensington Infirmary.

The Council of the Edith Cavell Homes of Rest for Nurses reports that during the year 582 nurses have been entertained. The houses now receiving guests are Coombe Head, Haslemere; the Crossways, Windermere; and the Hollies, West Norwood, of which the freehold is owned by the Fund. In addition, the Raven House, Adderley, has been placed at the disposition of the Fund by the Hon. Mrs. Corbett, M.B.E.; the Mythe Grange, Tewkesbury, is maintained at the expense of the same generous donor. Little Wych, Bridport; Oak Lodge, Hampstead; Winton House, Richmond; and Knightsbrook, Eastbourne, all of which were lent for a time now expired, have been closed. We note that the average cost for maintenance per guest per day was almost exactly 6s., but the statement is too vague to convey any useful information. It can only be surmised that it represents the average excess of expenditure per head per day over the payments made. It is very desirable that the amount contributed by guests towards their own maintenance should be specified, but we have not found this item in the accounts; the amounts entered as "Contributions from Guests," which are included in the "Local Income" of the Homes, totals only £132 for seven Homes, or an average of about 4s. 6d. per guest. The Homes are all conducted on a liberal scale, and the domestic arrangements and commissariat reflect the highest credit on the ladies in charge of the various establishments.

Lady Minto's Indian Nursing Association has had a successful year. A record number of nurses, thirty-three in all, were sent out, and the work has been much stimulated by a generous subscription through Lord Inchcape of £1,000 per annum for three years from the P. & O. Steam Navigation Company, on condition that all the nurses for India travel by this line and render assistance, on fees to be arranged, when called upon by the doctor on board. Notwithstanding this admirable arrangement, great difficulty was experienced in obtaining passages, and the increased cost of these makes a very serious demand on the Society's funds. A special selection committee, on which Miss Willcox, R.R.C., Matron of King's College Hospital, has consented to sit, interviews sisters whose applications have previously been passed by Miss Ray, R.R.C. Candidates must be fully-trained nurses between the ages of twenty-five and forty, with, as a rule, the C.M.B. certificate and experience in fever, tropical diseases, and private nursing, with massage. The pay begins at £90 a year, and increases by £5 a year to a maximum of £135, with everything provided and a yearly holiday allowance of £4 3s. 4d. The term of service to secure full outward and homeward passage, plus travelling allowance, is five years.

---

The Ramsgate General Hospital was *en fête* at the end of the year on the occasion of the unveiling by the Mayoress, Mrs. R. W. Philpott, of two brass tablets in commemoration of the benefits conferred upon the hospital by the voluntary war workers. One tablet records valuable help and financial gifts from the Kent V.A.D., G. F. Dabs, hon. treasurer, Florence Dunn, chairman, "and is a token of gratitude for the gift of £500 from the county funds, £104 from the local V.A.D. hospital, and valuable donations of dressings and equipment, probably worth from £400 to £500." The second tablet records the gift of £375 War Stock to the hospital by the voluntary workers at the Kinnaird House Canteen for H.M. Forces during the war. The Hospital Committee express themselves much indebted for the valuable help rendered since the war by the ambulance nurses, and also for the many hours' duty in the male wards which the men of the Brigade have undertaken. Miss Weigall, who was commandant at the Nethercourt Hospital, rendered a cordial tribute to the matron of the General Hospital, Miss A. Edgar, whose valuable help and advice was so freely given to the V.A.D. organisation. Wherever there is a spirit of friendship and reciprocity between voluntary hospitals and the V.A.D. workers there are sure to be openings for continued service, and we feel confident there will be room for V.A.D. developments at Ramsgate.

---

The growth of the South London Nursing Association has been phenomenal during the thirty-seven years it has been in existence. In the first year 225 patients were nursed, but last year the number rose to 3,178. This means that in twelve months the nurses had paid 40,706 visits to the homes of the patients. Besides that 32,924 children were attended at the London County Council centres. Naturally, with growth comes increased expenditure. In this direction the nurses have helped considerably. By means of collecting-cards they have gathered in £285 13s. 11d., whilst private cards and collecting-boxes realised £77 10s. 8d. We are not persuaded that it is the proper function of the nurses to collect. Wherever this is done there is danger of an assumption that the amount paid, usually a few pence, is due payment for services rendered, and this tends to cheapen the work of the nurses in the eyes of the patients.

---

We regret to learn that the Midwives' Institute is in sore straits as regards finance. An urgent appeal has been issued to members by the Honorary Treasurer, Miss Rosalind Paget, to attend the annual general meeting, which is to be held at the Society of Arts, John Street, Adelphi, on January 28, at 6 p.m., and take counsel as to the proper steps to be taken to rescue the situation. It appears that the income which used to suffice is now quite inadequate owing to large increases in rent, postage, stationery, heating, and other expenses. At least an extra £100 a year must be secured, and the prospects of raising this sum are not very bright. The Midwives' Institute cannot be spared without great loss to the midwives of the country. Midwifery is a very lonely career. It has many insidious difficulties and discouragements, which can best be met by maintaining a strong representative body, with officials competent to give expert advice, because chosen for their wide experience and sympathetic insight into members' trials. The teachers' instruction classes, inaugurated by the Institute last November and attended by seventy-six teachers, are a sign that the Council is alive to the need for raising the educational standard. It will be a calamity if this excellent beginning should fail to be carried further. During the past year there were 3,470 visitors at the offices and over 4,000 letters were sent out. Midwives should rally in great force on the 28th, and testify by an increase in their subscriptions their sense of Miss Paget's fine services on their behalf.

---

At the annual meeting of the Homesdale Cottage Hospital held before Christmas Lord Sackville presided, and made a presentation to the late matron, Mrs. Taylor, better known as Miss Siviour, who has lately completed a term of seventeen years in office. The gift consisted of a cheque for £160 together with an album containing the names of the subscribers. It was accompanied by a warm expression of appreciation for Mrs. Taylor's services. The newly appointed matron is Miss Baynes. Revised plans for the enlargement of the hospital to twenty-six beds are under consideration. The need for the enlargement is forcibly brought to notice by the fact that the operations have to be carried out in rooms at the Cornwall Hall, lent by the Wesleyan Church, Sister Bridges being in charge. Lord Sackville, strongly deprecating recourse to the rates for maintenance, announced a gift of £100 from a subscriber contingent on twenty more sums of the same amount being contributed to the building fund.

---

A local board of the Chartered Society of Massage and Medical Gymnastics is in course of formation at Manchester, where great interest and activity is exhibited by the members. In London a course on "Clinical Psychology," by Ernest Snowdon, M.B., will be given at the Armitage Hall, on the second and fourth Fridays of January, February, and March, at 6 p.m. The fee for the course is 10s., with transferable tickets. Lectures have been arranged at Bristol (Dr. Lily Baker), at Dublin, at Glasgow (Major Stevenson, of Bellahouston Hospital), and at Manchester (Dr. Langley), so that the year opens with considerable energy and should bring about a notable increase of membership.

## Figures and Tables

**Figure f1:**